# A GFP-Tagged Gross Deletion on Chromosome 1 Causes Malignant Peripheral Nerve Sheath Tumors and Carcinomas in Zebrafish

**DOI:** 10.1371/journal.pone.0145178

**Published:** 2015-12-22

**Authors:** Matteo Astone, Marco Pizzi, Margherita Peron, Alice Domenichini, Vincenza Guzzardo, Sonja Töchterle, Natascia Tiso, Massimo Rugge, Dirk Meyer, Francesco Argenton, Andrea Vettori

**Affiliations:** 1 Department of Biology, University of Padova, Padova, Italy; 2 General Pathology & Cytopathology Unit, Department of Medicine-DIMED, University of Padova, Padova, Italy; 3 Institute for Molecular Biology/ CMBI, University of Innsbruck, 6020 Innsbruck, Austria; National University of Singapore, SINGAPORE

## Abstract

Malignant peripheral nerve sheath tumors (MPNSTs) are highly aggressive soft-tissue sarcomas, characterized by complex karyotypes. The molecular bases of such malignancy are poorly understood and efficient targeted molecular therapies are currently lacking. Here we describe a novel zebrafish model of MPNSTs, represented by the transgenic mutant line *Tg(-8*.*5nkx2*.*2a*:*GFP)* ^*ia2*^. ia2 homozygous animals displayed embryonic lethality by 72 hpf, while the heterozygotes develop visible tumor masses with high frequency in adulthood. Histological and immunohistochemical examination revealed aggressive tumors with either mesenchymal or epithelial features. The former (54% of the cases) arose either in the abdominal cavity, or as intrathecal/intraspinal lesions and is composed of cytokeratin-negative spindle cells with fascicular/storiform growth pattern consistent with zebrafish MPNSTs. The second histotype was composed by polygonal or elongated cells, immunohistochemically positive for the pan-cytokeratin AE1/AE3. The overall histologic and immunohistochemical features were consistent with a malignant epithelial neoplasm of possible gastrointestinal/pancreatic origin. With an integrated approach, based on microsatellite (VNTR) and STS markers, we showed that ia2 insertion, in *Tg(-8*.*5nkx2*.*2a*:*GFP)* ^*ia2*^ embryos, is associated with a deletion of 15.2 Mb in the telomeric portion of chromosome 1. Interestingly, among ia2 deleted genes we identified the presence of the 40S ribosomal protein S6 gene that may be one of the possible drivers for the MPNSTs in ia2 mutants. Thanks to the peculiar features of zebrafish as animal model of human cancer (cellular and genomic similarity, transparency and prolificacy) and the GFP tag, the *Tg(-8*.*5nkx2*.*2a*:*GFP)* ^*ia2*^ line provides a manageable tool to study in vivo with high frequency MPNST biology and genetics, and to identify, in concert with the existing zebrafish MPNST models, conserved relevant mechanisms in zebrafish and human cancer development.

## Introduction

Zebrafish is gaining increasing relevance as animal model of human cancer, by providing both new insights in the field and powerful tools to carry out *in vivo* imaging, chemical and genetic screens, genetic and epigenetic modelling. Compared to other animal models, its specific features make zebrafish easier to manage high numbers of individuals, genetic manipulations and analysis of embryonic cancer-related phenotypes and adult tumors. The significance of zebrafish as cancer model is given by the fact that these aspects couple with the strong resemblance of zebrafish cancers with their human counterpart at the histological, gene expression and genomic levels [1,2].

Malignant peripheral nerve sheath tumors (MPNSTs) account for 5–10% of all soft-tissue sarcomas and usually arise from peripheral nerves. In humans they occur sporadically or associated with neurofibromatosis type 1, representing the leading cause of mortality in this disease. MPNSTs mainly affect adults and appear earlier in patients with neurofibromatosis type 1 [3]. The high recurrence rate (up to 40%), the tendency to metastasize (two-thirds of the cases) and the limited sensitivity to chemo and radiation therapy make MPNSTs highly aggressive tumors with a poor prognosis. Moreover, effective targeted molecular therapies are currently not available and surgical resection remains the treatment of choice, often accompanied by chemotherapy and radiation therapy [4,5].

MPNSTs have complex karyotypes, with massive aneuploidy and heterogeneity [5,6]. Loss of *NF1* and *p53* are the most frequent gene alterations, and the majority of MPNSTs show a gene expression signature indicating p53 inactivation [5,7]. Nevertheless, the molecular bases of this malignant transformation are still poorly understood. Specific regions of copy number alteration have been found associated with poor patient survival [8], but the work for the identification of candidate genes driving MPNSTs carcinogenesis is just at the beginning [5,6].

In zebrafish, malignant neoplasms resembling human MPNST have been described in a number of mutant lines and designated as zMPNST (zebrafish MPNSTs). Inactivating mutations have been reported in 17 of 28 ribosomal protein (*rp*) genes, in *tp53*, in 3 major mismatch repair (*mmr*) genes and in *NF2a* and predisposes zebrafish to MPNSTs [9,10,11,12]. In MPNSTs derived from zebrafish lines with *rp* gene mutations, a loss of p53 synthesis was reported, despite the presence of a wild-type *tp53* gene [13]. Moreover, zMPNSTs arising in fish heterozygous for *rp* or homozygous for a *tp53* mutation are highly aneuploid. This feature resembles human MPNSTs, and is not shared by most murine cancer models [6]. For all these reasons, zebrafish turned out to be a promising animal model to unravel the molecular basis of MPNST biology and to identify important drivers in human cancer.

Here we report the characterization of a novel zebrafish model of MPNST, represented by the transgenic mutant line *Tg(-8*.*5nkx2*.*2a*:*GFP)* ^*ia2*^ and defined by the presence of a gross deletion in chromosome 1. The deletion is embryonic lethal in homozygotes and developmentally inconsequential in heterozygotes. Importantly, we observed the development of zMPNSTs and abdominal carcinomas with high frequency in adult *Tg(-8*.*5nkx2*.*2a*:*GFP)* ^*ia2*^ fish.

## Results

### Homozygous *ia2* mutant fish die during embryonic development

**Fig 1:**
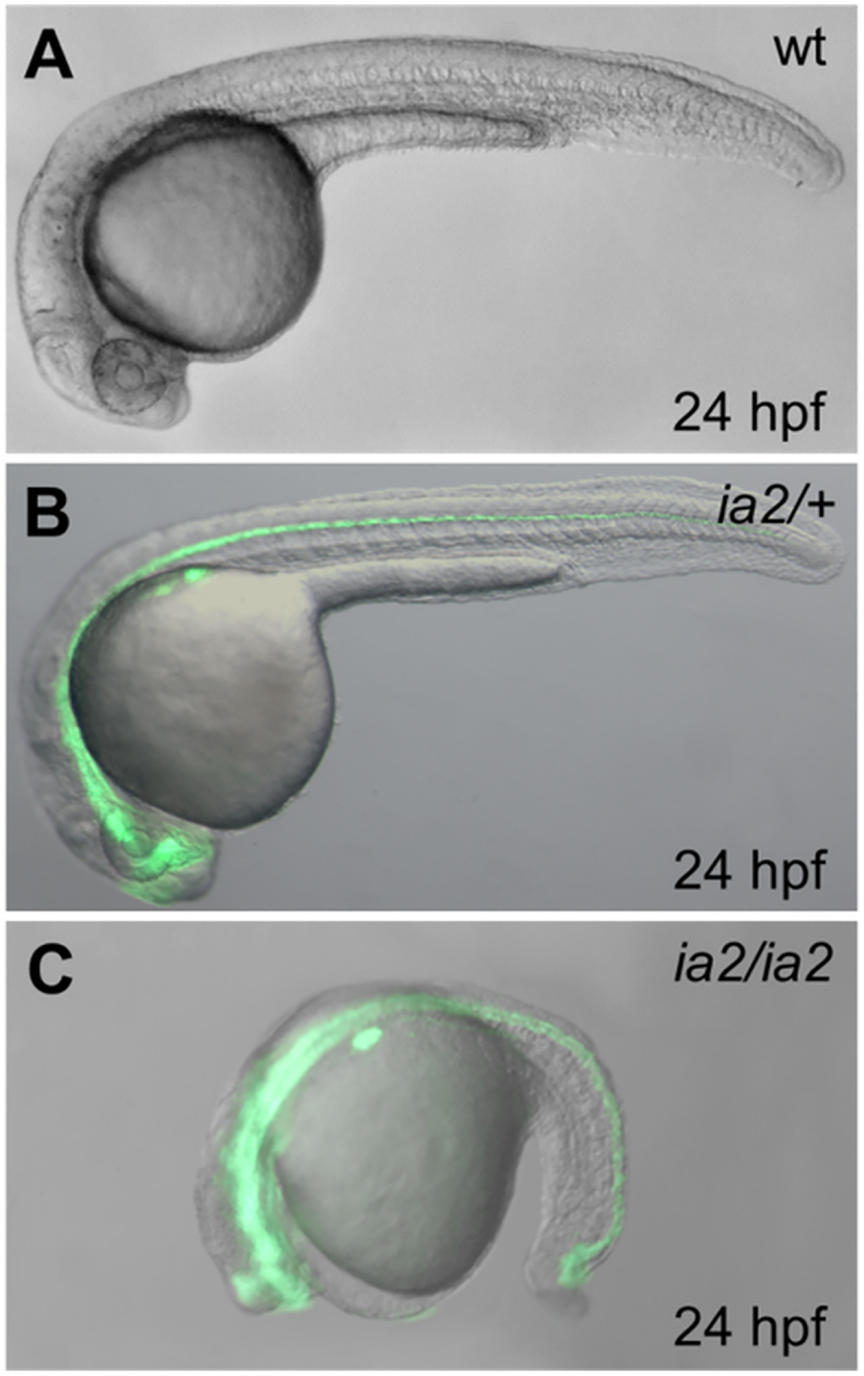
Phenotype of the ia2 mutant at 24 hpf. The homozygous mutant (C) displays a strongly delayed development and compromised phenotype, while the heterozygote (B) appears indistinguishable from the wild-type (A). The green fluorescence relative to the *-8*.*5nkx2*.*2a*:*GFP* transgene is present in heterozygous and homozygous ia2 mutant fish, being the *GFP* gene co-inherited with the ia2 mutant allele. This allows the early and immediate screening of the three genotypes.

*Tg(-8*.*5nkx2*.*2a*:*GFP)* ^*ia2*^ is a transgenic fish line expressing the GFP under the control of a 8.5 kb-long *nkx2*.*2a* gene promoter fragment. This line was generated to explore the role of Nkx2.2a transcription factor in pancreas development [14]. In an attempt to generate homozygous transgenic fish, we observed that the 25% of the offspring obtained by the incross of two heterozygous *Tg(-8*.*5nkx2*.*2a*:*GFP)* ^*ia2*^ fish were not viable by 72 hpf (Fig 1). Moreover, the fluorescent fish with a normal embryonic phenotype (*Tg(-8*.*5nkx2*.*2a*:*GFP)* ^*+/ia2*^ *)* develop tumor masses in adulthood with high frequency.

**Fig 2:**
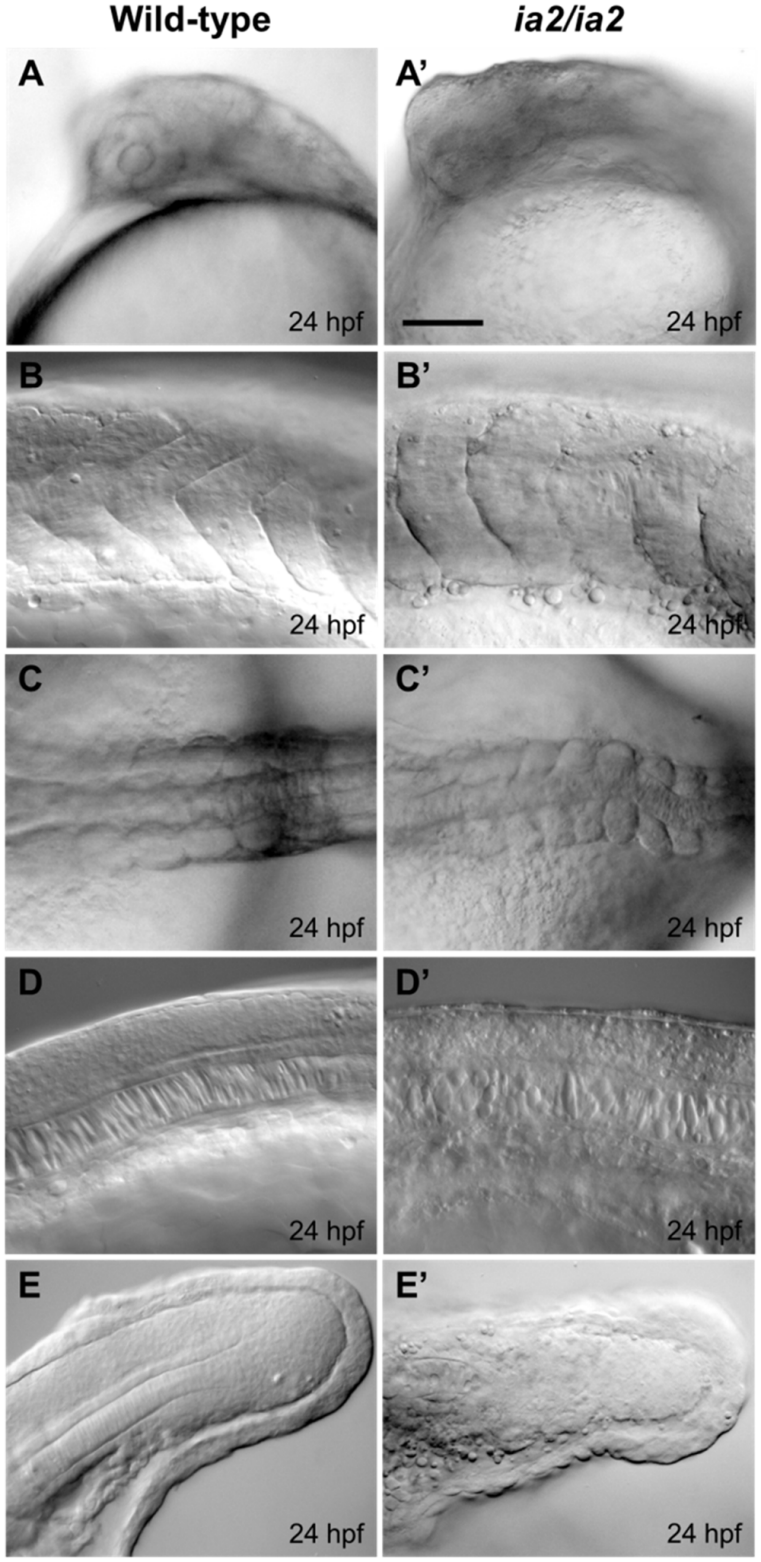
Morphological alterations of ia2/ia2 mutant at 24 hpf. (A, A’) Head side view: mutant embryos display a relevant delay in head structures development, with just sketchy eye, when visible. (B, B’) Somites side view, showing their poorly defined shape and boundaries in the mutant. Globular cells with impaired adhesion are also visible. (C, C’) Notochord dorsal view: in mutant embryos notochord bending on the coronal plane is evident. (D, D’) Notochord side view, showing how notochord cells structure is also affected in mutant embryos, with globular and disorganized vacuoles, compared to the stretched and flanked ones of the wild-type. (E, E’) Tail terminal portion, side view: the notochord is indistinguishable in the mutant and the mesenchyme seems disaggregated with many non-adherent globular cells. Scale bar 50 μm.

Microscopic *in vivo* evaluations of ia2/ia2 mutant embryos reveals harsh morphological alterations already visible at 15 somites stage. At 24 hpf their development is markedly delayed (Fig 2) and almost arrested, especially at the level of the head, then they only elongate in body axis until embryonic lethality appears. At 24 hpf the head of ia2/ia2 embryos still resembles that of wild-type embryos at 13 somites stage: the eye, when visible, is sketchy and the lens are absent (Fig 2A and 2A’). The somites lose their characteristic V shape, and the metameric structure of the paraxial mesoderm looks less defined when compared to wild-type; the somite boundaries are undefined and show globular cells with impaired adhesion (Fig 2B and 2B’). ia2/ia2 embryos display notochord bending, both in sagittal and coronal planes (Fig 2C and 2C’). By using notochord-specific markers, such as *notail*, alterations are already detectable at 7 somites stage (S1A–S1D’ Fig). Notochord cells are also affected: the vacuoles appear globular and disorganized, a typical condition of early notochord developmental stages (Fig 2D and 2D’). The tail bud shows a strongly disaggregated mesenchyme, without distinguishable notochord precursors and with the presence of many non-adherent globular cells (Fig 2E and 2E’). At 30 hpf, also the nervous and muscular systems result to be altered. As revealed by immunofluorescence experiments, ia2/ia2 embryos are characterized by a disorganized axonal network and by a dramatic reduction of muscle fibers mass (S1E–S1H Fig). Moreover, cell death is massive at the level of the head and of the terminal portion of the tail (S1I and S1L Fig).

### Mutant ia2/+ fish develop zMPNSTs and abdominal carcinomas

**Fig 3:**
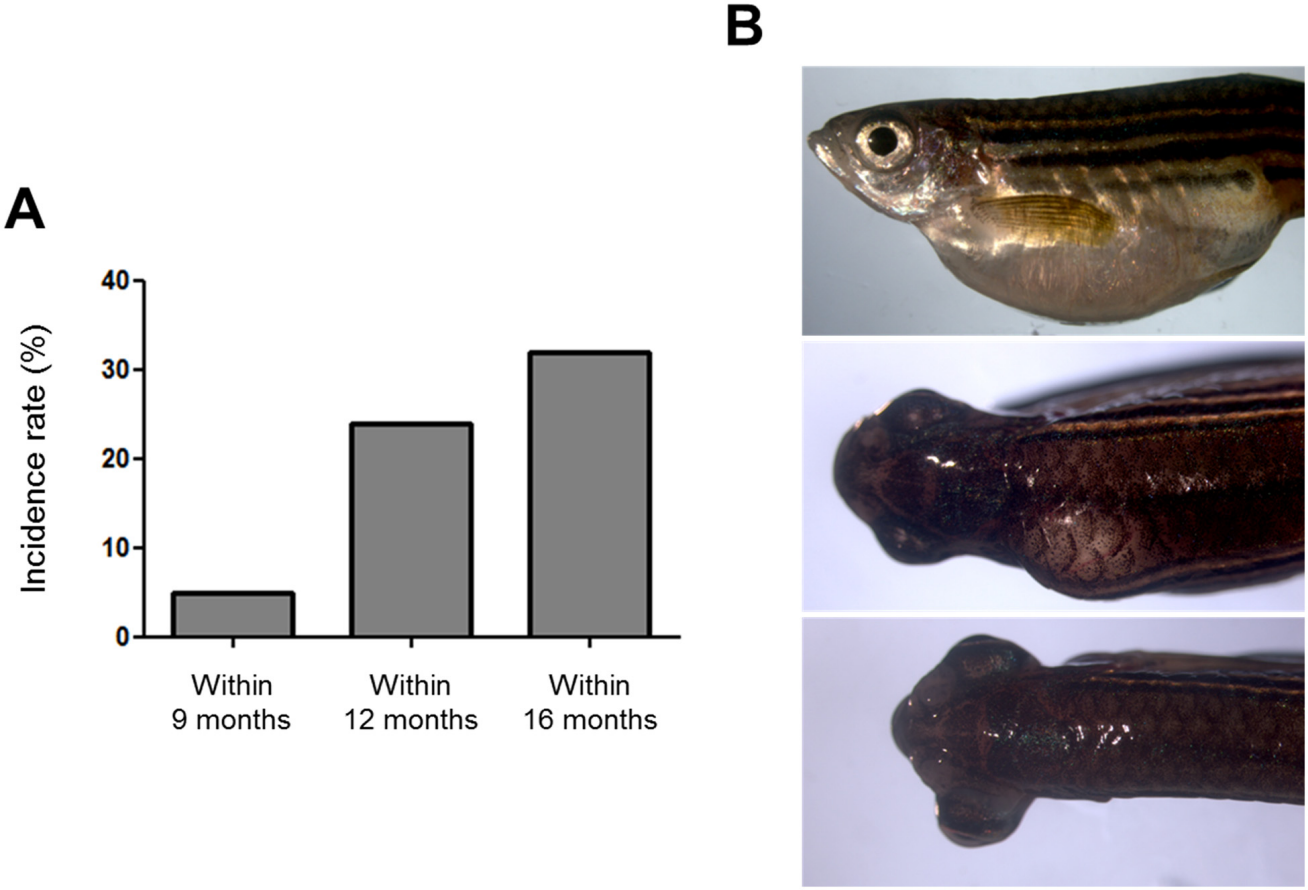
Incidence rate tumors in heterozygous ia2 mutant (ia2/+). (A) Cumulative percentages of tumor masses developed by ia2/+ individuals within 9, 12 and 16 months of age are depicted. (B) Representative images of the spontaneous tumor masses developed by heterozygous ia2/+ mutants, arising in the abdomen (top), along the spinal cord (middle) and intrathecally, causing the lateral projection of the eye (bottom).

*In vivo* microscopic evaluation and other analyses performed on ia2/ia2 homozygous mutants could not reveal alterations in ia2 heterozygous embryos and larvae that appear indistinguishable from the wild-type (Fig 1). However, ia2/*+* fish develop visible tumor masses with an incidence rate of 10% by 9 months, 24% by 12 and 32% by 16 (Fig 3A). The majority of ia2 masses appear as a huge swelling of the abdomen. Less frequently, the masses arise dorsally, along the spinal cord, or intrathecally, causing the lateral extrusion of the eye (Fig 3B). A first histological evaluation of such lesions suggested the occurrence of at least two different neoplastic histotypes, with either epithelial or mesenchymal features. All the tumors analyzed disclosed overt malignant features, such as invasive growth pattern, high grade cytological atypia, necrosis and evident mitoses.

**Fig 4:**
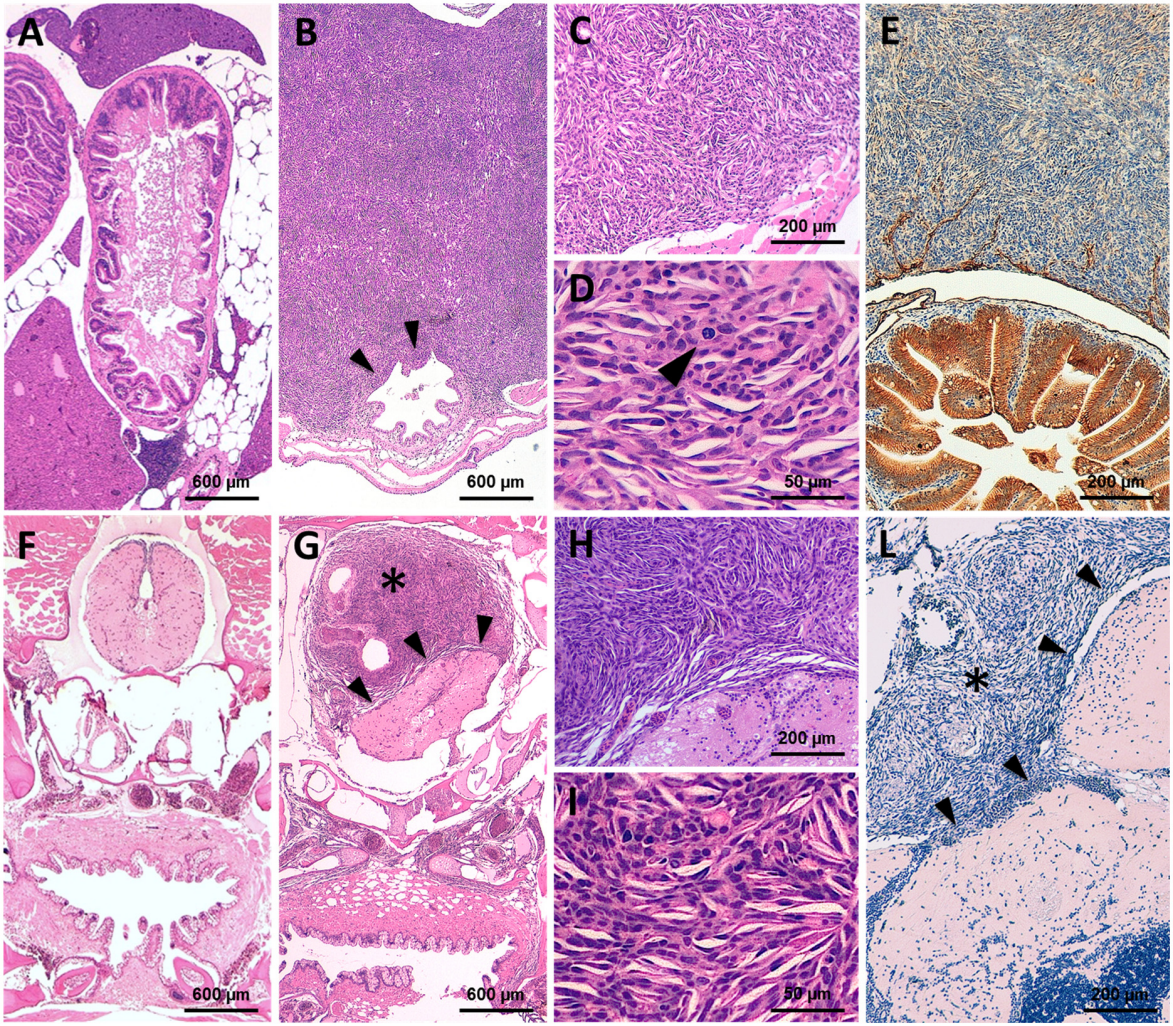
Representative histological features of the ia2 MPNSTs. (A) In normal controls (i.e. non-neoplastic cases), transverse sections of the abdominal cavity show the gastrointestinal tract (center), surrounded by pancreatic and adipose tissue. The liver (top center and lower left) occupies more peripheral regions. (B-E) Abdominal MPNST. (B) The tumor occupies the abdominal cavity under the swim bladder and infiltrates the intestinal wall (arrowheads). (C, D) Cytologically, the lesion is composed of spindle to epithelioid cells with high nuclear-to-cytoplasm ratio, clumped chromatin and evident mitoses (arrowhead in D). (E) The intra-abdominal MPNSTs stains negative for AE1/AE3 (positive internal control: intestinal epithelial cells, stained in brown). (F) In non-neoplastic cases, transverse sections of the cranial region display the oral cavity/pharynx (bottom center), lying beneath the basicranial structures (middle center) and the brain stem (top center). (G-L) Intrathecal MPNST. (G) The intrathecal neoplastic mass (star) compresses and displaces the adjacent medulla oblongata (arrowheads), without clear-cut infiltration of the neural parenchyma. (H, I) Cytologically, the lesion closely resembles its intra-abdominal counterpart. (L) Pan-cytokeratin immunostain is also negative in intrathecal MPNST (star). Arrowheads: tumor/brain stem interface.

Twenty-six of 48 fish with visible masses (54.2%) presented spindle cell neoplastic lesions with peculiar anatomical and histopathological features. They arose either in the abdominal cavity (19/26 cases) or as intrathecal/intraspinal lesions (7/26), with dislocation and infiltration of the surrounding anatomical structures (Fig 4). The neoplastic population was composed by spindle to fusiform cells, with fascicular and storiform growth pattern. Microfoci of necrosis or large necrotic areas were also commonly observed. Large epithelioid cells were occasionally faced, but definite epithelial differentiation was never present (Fig 4C, 4D, 4H and 4I). All these tumors stained negative for cytokeratin AE1/AE3 (Fig 4E and 4L). The overall histologic and immunohistochemical features showed a high grade soft-tissue sarcoma, consistent with the zebrafish malignant peripheral nerve sheath tumors (zMPNSTs), as described by Amsterdam and colleagues [15].

**Fig 5:**
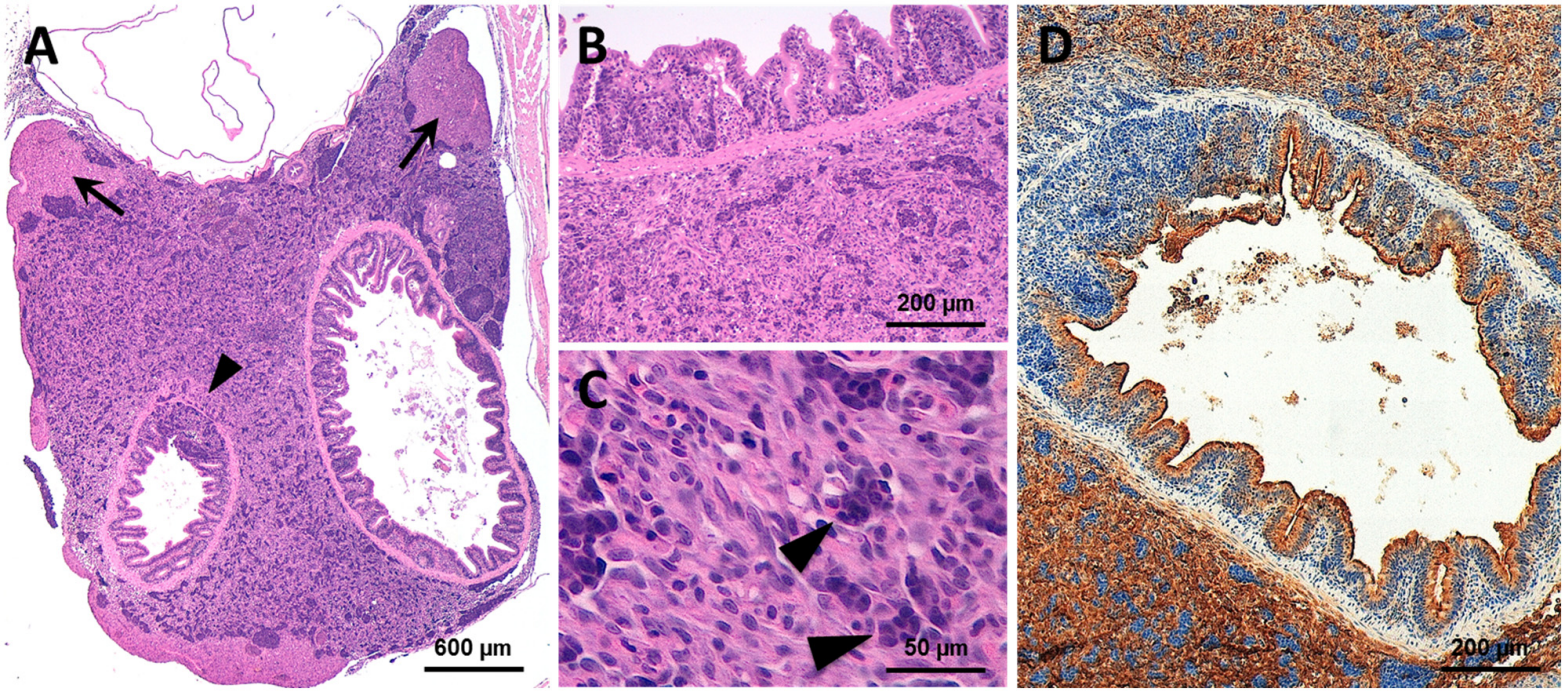
Representative histological features of the ia2 abdominal carcinomas. (A) Abdominal tumors arise beneath the swim bladder, in close proximity with the gut, pancreas and liver. The neoplastic cells occasionally infiltrate the hepatic parenchyma (arrows) and the intestinal wall (arrowhead). (B, C) Cytologically, the tumor is composed of polygonal cells with abundant eosinophilic cytoplasm. In most cases, a sub-population of epithelioid cells with more basophil cytoplasm is also faced (arrow). (D) The abdominal carcinomas consistently express the pan-cytokeratin AE1/AE3 (brown staining). See Fig 4A for the normal abdominal region.

The remaining 22 of the 48 analyzed fish (45.8%) displayed tumor masses with significantly different macroscopic and histologic features. These neoplastic lesions disclosed a solid growth pattern, with fascicles, nests and cords of highly atypical cells. They invariably occupied the abdominal cavity beneath the swim bladder, displacing and infiltrating the intestinal wall and the liver (Fig 5A). Cytological examination disclosed the presence of polygonal to elongated cells, with increased nuclear to cytoplasmic ratio and prominent nucleoli. The cytoplasm varied from scant and basophilic to fairly abundant and eosinophilic (Fig 5B and 5C). The immunohistochemical staining for cytokeratin AE1/AE3 disclosed a sharp positivity in a proportion of neoplastic cells (Fig 5D). The pathological features suggested a malignant neoplasm with epithelial features. The anatomical location and the infiltration pattern were compatible with a carcinoma of gastrointestinal and/or pancreatic origin.

### ia2 zebrafish mutant line is characterized by a gross deletion on chromosome 1

To understand the genetic cause of ia2 mutant phenotype, we decided to map the mutation in the zebrafish genome. With this aim a genome-wide linkage analysis was carried out using a panel of 288 polymorphic microsatellite markers, evenly spaced along the zebrafish chromosomes. The first screenings were performed by comparing a pool of wild-type with a pool of ia2/ia2 homozygous mutant embryos. Selection and identification of these two groups was performed considering that wild-type embryos do not express the GFP, while ia2 mutant embryos are easily recognizable by their peculiar phenotype (Fig 1). Out of the 288 microsatellite markers analyzed for each pool, only the microsatellites Z7353 and Z1351 on chromosome 1 resulted to show a possible association with the ia2 mutation. We therefore focused our attention on this chromosome, increasing the number of markers analyzed and enlarging the sample of ia2 embryos. In particular, for this second screening we selected additional markers belonging to the Shimoda panel [16] and new polymorphic microsatellites specifically identified for this study in our laboratory (S1 Table). By single-embryo genotyping, markers Z7353 (at 30.40 Mb), Z1351 (at 33.17 Mb) and Z10124 (at 35.08 Mb) exhibited very few recombination events in ia2 mutants. We never observed recombination events for markers Z10978, Z6403, Z6411 and zK59P5, thus confirming the strong association between the ia2 mutation and these microsatellites located on chromosome 1.

**Fig 6:**
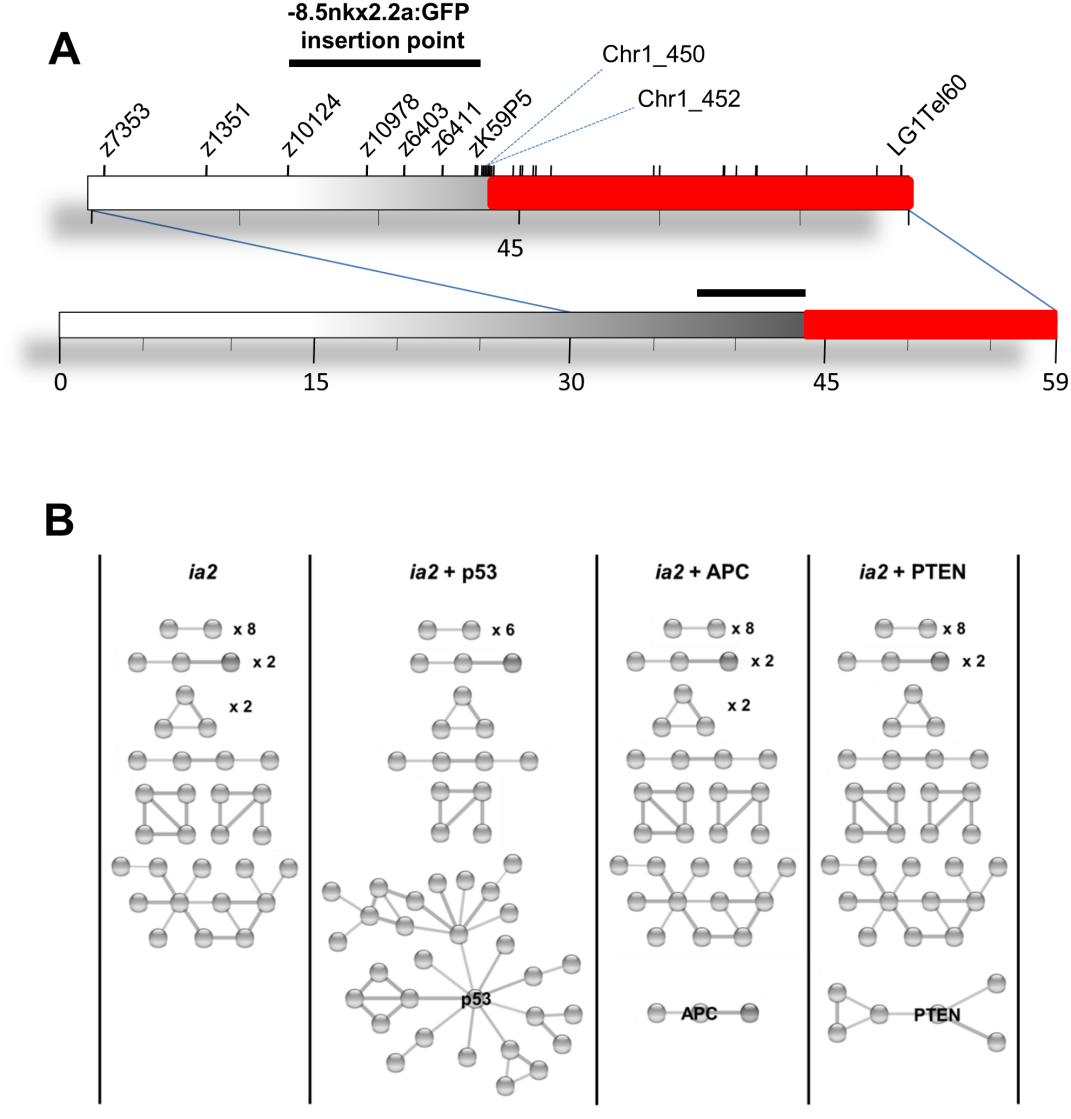
Identification and analysis of ia2 deletion. (A) Schematic representation of ia2 deletion in chromosome 1 (in red). All the STS and genes mapping in the region defined by marker chr1_452 (272 bp downstream Chr1_ 450) and LG1Tel6035 were not amplifiable by PCR in homozygous mutants. According with marker positions, we identified in chromosome 1 of *Tg(-8*.*5nkx2*.*2a*:*GFP)* ^*ia2*^ embryos a deletion of 15.2 Mb. The black bar displays the position where the -8.5nkx2.2a:GFP transgene was mapped by linkage analysis. (B) STRING analysis of the protein-protein interactions among *p53* and the products encoded by the zebrafish ia2 deleted genes. A first STRING analysis was performed on the products encoded by the human orthologues of the ia2 deleted genes only (ia2). The virtual addition of p53 determines the formation of a big cluster, with p53 directly linked with 9 ia2 deleted gene products (ia2 + p53). Other important tumor suppressors don’t share the same feature: as an example, the analysis with APC (ia2 + APC) and PTEN (ia2 + PTEN) are reported. Disconnected nodes are not shown.

Surprisingly, trying to analyze other markers to better localize the ia2 mutation, we realized that all the microsatellite from zC243A20_A (44.66 Mb) to the end of telomere, were not amplifiable by PCR in homozygous mutants, while in wild-type and heterozygous siblings the expected PCR product was detectable. These results let us establish that ia2 mutants are characterized by the presence of a deletion in chromosome 1, presumably generated during the integration process of *-8*.*5nkx2*.*2a*:*GFP* transgene. For this reason, considering that the ia2 deletion is apparently co-inherited with the ia2 transgene, we also asked whether the presence of the GFP was strictly associated with ia2 mutation, as initially assumed. As expected, by linkage analysis we identified 4 microsatellites flanking the ia2 deletion that strongly associated with the GFP. This is confirmed by the statistically significant LOD score value, calculated between microsatellites Z10978, Z6403, Z641 and zK59P5 (maximum multipoint pLOD score of 101.0, at a recombination fraction of 0.00; p = 1.84E-103). According to the recombination events analysis and LOD score data, the ia2 transgene is located in a region of chromosome 1 between marker Z10124 (35.08 Mb) and the beginning of the ia2 deletion (Fig 6A).

Finally, to precisely identify the dimension of ia2 deletion and the position where the chromosome breaking occurred, 32 additional STS (sequence-tagged site) markers were analysed by single embryo PCR (S1 Table). With these experiments, we established that the ia2 mutation is a deletion of 15.2 Mb spanning from marker chr1_450 at 43.69 Mb to the end of chromosome 1, represented by the telomeric microsatellite marker ZLG1Tel6035 at 58.88 Mb (Fig 6A).

According with Genome Browser database (assembly GRCz10/danRer10), ia2 deletion involves 241 genes mapping in the telomeric region of chromosome 1 (S2 Table). All these genes were submitted to the PANTHER classification system and analyzed according to the biological process class of the products encoded. We then considered those biological processes implicated in cancer hallmarks [17]. Among ia2 deleted genes, 9 are involved in apoptosis, 10 in cell adhesion, 12 in cell cycle, 4 in energy metabolism and 25 in immune response (S3 Table), thus representing candidates in ia2 carcinogenesis.

Concerning MPNSTs in particular, it was shown in zebrafish that mutations in *p53*, *nf2a*, mismatch repair (*mmr*) and ribosomal proteins (*rp*) genes lead to the development of this tumor type [10,11,12,15]. We therefore wondered if these genes were present in ia2 deletion, thus suggesting possible drivers for the carcinogenesis of ia2 mutant. Interestingly, *rps6* gene, mapping within the ia2 deletion, codes for the 40S ribosomal protein S6 (RefSeq zgc:92237) (S2 Table), that belongs to the same class of ribosomal proteins known to be associated with MPNSTs development [15]. In addition, MPNSTs arisen in all zebrafish *rp* mutants display an impairment in p53 protein synthesis [10,13]. Although in ia2 mutants *tp53* is not deleted, according to STRING database, p53 is directly correlated with 9 (*aptx*, *acta1*, *slk*, *rad23a*, *mdh1*, *taf5*, *map2k7*, *dnajb1*, *jag1*) of the ia2 deleted gene products, forming the biggest protein cluster resulting from the STRING database analysis. Worth noting, this clustering ability of p53 is not shared by other important tumor suppressors, such as APC, PTEN, RB, SMAD4, VHL, BRCA1/2 (Fig 6B).

## Discussion

In this study, we presented a zebrafish mutant line named *Tg(-8*.*5nkx2*.*2a*:*GFP)* ^*ia2*^, characterized by a 15.2 Mb deletion affecting 241 genes in chromosome 1. Considering that the fluorescence of the transgene, after more than 10 outcrosses, is still inherited by 50% of the offspring of each ia2 heterozygous carrier, we can definitely conclude that the transgene has only one site of integration. Moreover, the fluorescence of the transgene and the deletion were co-inherited in all cases examined, hence, we infer that the deletion is possibly due to the insertional alteration caused by the random integration of *8*.*5nkx2*.*2a*:*GFP* transgene [14]. In homozygosis, the deletion leads to a severe phenotype in early development and is embryonic lethal by 72 hpf. Surprisingly, ia2 heterozygotes have no overt developmental defects despite the hemizygosity of such a great number of genes, meaning that a single dose for each of the genes in the ia2 deleted region is sufficient for survival and reproduction. This is fitting with the viability of animals with large deletions obtained by gamma-ray screenings of mutations [16], hence the idea that haploinsufficiency, (i.e. insufficient production of a protein from a single allele that causes a dominant genetic defects) can be considered a quite rare event in zebrafish. At this regard, ia2 mutant could represent a tool to study the genotype/phenotype correlation in allelic collections of hypomorphic mutants that can be easily generated by CRISPR/Cas technology for genes belonging to ia2 deleted region or for the presence of enhancers or suppressors in the same region. ia2 mutants can also be used to map any loss of function mutation in the ia2 deleted region by a simple complementation test. With regard to these applications as well as to the study of ia2 tumorigenic phenotype itself, it is worth noting that the GFP gene being co-inherited with ia2 mutation, enables a rapid and very early screening of ia2 carriers without the need of any genotyping step, a feature not present in large multigene deletion mutants obtained with gamma-ray [16].

The characteristic phenotype of ia2 mutant is the development of spontaneous tumors in the adult heterozygous carriers. According to the histologic features, the tumors fall into two categories that are almost equally frequent: (i) carcinomas of probable gastrointestinal/pancreatic origin and (ii) soft-tissue sarcomas. The latter were most consistent with zMPNSTs, described in zebrafish lines carrying mutations of different ribosomal protein genes, *p53* and three *mmr* genes mutants [10,11,12,15]. The peculiar anatomical regions in which ia2-associated zMPNSTs arise (abdominal and intrathecal/intraspinal districts) match with previously described models. The overall histologic and immunohistochemical features further confirm the tumor histotype [10,11,15].

The precise genetic mechanism of ia2-induced carcinogenesis still remains elusive. One possibility is that the exogenous enhancer of *nkx2*.*2a* is activating an oncogene. In that case, however, we would expect the tumors to be fluorescent because of the activity of the same enhancer on the GFP gene, which is in *cis*; notably, the ia2 tumors aren’t fluorescent. A second possibility is the transgene to be inserted in a single tumor suppressor gene, located between markers Z10124 and chr1_450. On the other hand, it is reasonable to believe the 15.2 Mb deletion in chromosome 1 to be the cause of the tumors in ia2 fish, due to the large number of deleted genes that might play a role in cancer development.

The ontology analysis carried out with PANTHER classification systems supports this hypothesis, showing that several ia2 deleted genes can be associated with biological processes implicated in cancer development, such as apoptosis, cell adhesion, cell cycle, energy metabolism and immune response (S3 Table). These observations may also explain why the same genetic aberration can lead to different tumor histotypes. Different combinations of carcinogenic mechanisms may indeed take part in variable cell types, thus leading to the development of different ia2-induced tumor histotypes.

Moreover, the analysis of ia2 deleted genes suggested some interesting considerations regarding ia2 zMPNST pathogenesis. None of the genes previously shown to be causative of zMPNST after inactivating mutations [10,11,12,15] are deleted in ia2 mutant, ruling out the hypothesis of a direct inactivation of one of those alleles responsible for the same tumor histotype in the existing MPNSTs zebrafish models. However, the deletion in ia2 of the *rps6* gene, that encodes the 40S Ribosomal Protein S6, indicates an interesting insight. Lai and colleagues [12] showed that 17 of 28 zebrafish heterozygous mutant lines characterized by the loss of function of 28 different rp genes (*rps6* was not included) developed zMPNSTs, and suggested that many rp genes act as haploinsufficient tumor suppressors in fish. This, together with our finding, presented *rps6* gene as one of the possible drivers for the zMPNSTs developed by ia2 mutant. Despite the width of the deletion, 54.2% of ia2 tumors are specifically represented by zMPNSTs, suggesting that the *rps6* gene haploinsufficiency might be the initial driver biasing the tumor histotype.

The *rp* mutants described by Amsterdam et al. [15] display a loss of p53 synthesis in MPNST cells [13] and p53 is certainly involved in zMPNST development, since heterozygous *p53* inactivating mutations are sufficient for zMPNST appearance [18]. Protein-protein interaction analysis based on the STRING database, shows that p53 directly interact with many products encoded by genes deleted in ia2. An involvement of p53 in MPNSTs pathogenesis might therefore represent a feature shared also by MPNSTs developed in ia2 mutants.

The high level of aneuploidy observed in human and zebrafish MPNSTs is independent of the original genetic alteration; however, common regions of CNVs were identified in any given zMPNSTs [6]. Thus, ia2 represents a new MPNST animal model that enriches the possibilities to study MPNST biology and to identify conserved biologically relevant drivers in zebrafish and human cancer.

Even though further studies are required to better define ia2 zMPNSTs pathogenesis and the tumor histotype of the epithelial malignancies, the ia2 mutant is a manageable model that takes advantage of the GFP expression to study both MPNST and the collection of genes in the ia2 deleted region.

## Materials and Methods

### Animals

Animals were staged and fed as described by Kimmel et al.[19]. The project was examined and approved by the Ethics Committee of the University of Padua with protocol number 18746. The mutant carriers of *Tg(-8*.*5nkx2*.*2a*:*GFP)* ^*ia2*^ transgenic line, already described in Pauls et al. [14] were identified by the GFP expression (heterozygotes and homozygotes) and the phenotype (homozygotes). Fish of all strains in Padova Zebrafish facility are monitored daily for the presence of signs of sickness, pain, distress, suffering, or moribund conditions; all affected fish are euthanized, beheaded and analyzed.

### Whole-mount *in situ* hybridization and immunohistochemistry

Whole mount RNA *in situ* hybridization was performed as described before [20]. *notail* probe was synthetized using DIG-labeled ribonucleotides, T7 RNA polymerase and *Xho*I-linearized pBSIISK1. For the anti-acetylated tubulin immunostaining, embryos were fixed in 4% PFA/PBS overnight and then stored in 100% methanol at -20°C. Embryos were permeated through incubation with acetone at -20°C, rehydrated in PBS and washed in 1% TritonX-100 in PBS (PBTX). They were immunostained with antibody anti-acetylated tubulin (T7451, Sigma-Aldrich) and the anti-mouse immunoglobulins/TRITC (R0270, Dako, Glostrup, Denmark) secondary antibody, according to standard procedures.

### Rhodamine-phalloidin and acridine orange staining

For the rhodamine-phalloidin staining, embryos fixed in 4% PFA/PBS were permeated through incubation with PBTX 2% O/N at 4°C. Blocking was done with 1% DMSO, 1% BSA in PBS for 30 minutes at room temperature. Embryos were stained through incubation with phalloidin-TRITC (P1951, Sigma-Aldrich) 1:1000 in PBTX 1%. For acridine orange staining, alive embryos were incubated with 15 μg/ml acridine orange (A6014, Sigma-Aldrich) for 30 minutes at 28,5°C, washed twice in fish water and examined immediately after.

### Microscopy and image acquisition

GFP-expressing embryos and acridine orange-stained embryos were analysed using a Leica M165FC epifluorescent microscope. *In situ* hybridizations were documented under a Leica DMR microscope. All pictures were acquired with a Leica DC 500 digital camera and contrast and brightness elaborated with Adobe Photoshop 6.0 software. Confocal images were acquired with a Bio-Rad radiance 2000 confocal system.

### Histological and immunohistochemical evaluation of zebrafish tumors

Overall, 54 zebrafish were submitted for histological and immunohistochemical evaluation: (i) 41 cases with abdominal tumors; (ii) 7 cases with ocular/head tumors; (iii) 6 negative controls (3 males and 3 females, with no macroscopic evidence of disease).

The morphological evaluation was performed on formalin-fixed, paraffin-included tissue samples. Slides were stained with H&E for morphological evaluation. Immunohistochemical analysis was performed on 4 μm-thick sections, using the anti-pan cytokeratin monoclonal antibody AE1/AE3 (ab961, Abcam Cambridge-UK). Enzymatic retrieval was applied and antigen detection was performed in an automated immunostainer (BOND-MAX, Leica Biosystems, Milan-Italy), as previously described [21].

### Genome-wide linkage analysis

*Tg(-8*.*5nkx2*.*2a*:*GFP)* ^*ia2*^ heterozygous fish were outcrossed to wild-type individuals of the Tübingen strain. F1 heterozygous mutants were raised and intercrossed. F2 homozygous mutant embryos (ia2/ia2) and wild-type siblings were identified as described before, collected and fixed in 4% PFA/PBS at 30 hpf. A genome-wide linkage analysis was performed using a panel of 288 polymorphic microsatellite markers (primer sequences and map positions are available upon request to the authors). The first screening was carried out on genomic DNA extracted from a pool of 42 ia2/ia2 embryos and a pool of 36 wild-type siblings according to standard procedures. Single-embryo DNA for the subsequent analysis was extracted by incubation with proteinase K 1 mg/ml in ELB lysis buffer (10 mM Tris pH 8,3; 50 mM KCl; 0,3% Tween-20; 0,3% NP40) for 4 h at 55°C, followed by inactivation at 94°C for 10 minutes. Single-embryo PCR was performed for Z7353 and Z1351 markers of the previous panel, for the genes and the new microsatellites listed in S1 Table and for the following markers belonging to the Shimoda panel (Shimoda et al., 1999): Z10194, Z1705, Z9958, Z1436, Z9125, Z9042, Z10124, Z23059, Z10978, Z11859, Z6403, Z6411, Z21548, Z11154, Z10888, Z1173, Z8678, Z13724. To identify the position of *-8*.*5nkx2*.*2a*:*GFP* transgene insertion, parametric LOD score was calculated using GeneHunter software. Linkage analysis was performed assuming a dominant model with a complete penetrance. To assess the statistical significance of linkage, we considered the classical threshold value of 3.0.

### Software and databases

The list of ia2 mutant deleted genes was produced by using Genome Browser database (http://genome.ucsc.edu/cgi-bin/hgGateway), assembly GRCz10/danRer10. The list was submitted to the PANTHER classification system (http://www.pantherdb.org) for the ontology analysis. Human orthologues of zebrafish ia2 mutant deleted genes were obtained with ZebrafishMine (http://www.zebrafishmine.org/begin.do) and submitted to STRING database (http://string-db.org/) for the protein-protein interaction analysis, with a required confidence of 0.6. The same analysis was repeated by adding one by one the following tumor-suppressors genes: *p53*, *APC*, *PTEN*, *RB*, *SMAD4*, *VHL*, *BRCA1/2*.

## Supporting Information

S1 Fig Phenotype of ia2/ia2 mutant. (A-D) Notochord alterations are evidenced by *in situ* hybridization for *notail* already at 7 somites stage. Mutant notochord is shorter and wider with respect to the wild-type (C, C’). Notochord bending (A-B’) and the morphological abnormalities of notochord structure and vacuolated cells (C-D’) are also apparent. (E, F) Axonal microtubules staining with anti-acetylated tubulin antibody. Confocal Z-stack projection; trunk side view. The mutant shows a highly disorganized axonal network. (G, H) Rhodamine-phalloidin staining. Confocal Z-stack projection; trunk side view. In the mutant the muscle fibers are strongly reduced in number, with an overall dramatic decline in muscle fibers mass. (I, L) Acridine orange staining. Massive cell death is evident in ia2/ia2 mutant, particularly in the regions of the head and the tail corresponding to the morphologically most altered districts. The intense GFP fluorescence of the neural tube visible in the mutant is that characteristic of the *Tg(-8*.*5nkx2*.*2a*:*GFP)* ^*ia2*^ transgenic line.
(TIF)

S1 Table New microsatellite markers and genes used to define *ia2* deletion. The position in Mb in chromosome 1 and the primers designed are reported. Deleted STS markers are reported in bold.
(TIF)

S2 Table List of genes deleted in ia2 mutant. (PDF)

S3 Table PANTHER classification of ia2 deleted genes belonging to biological process classes implicated in cancer. (TIF)
