# Supplementary figures and images for: A GFP-Tagged Gross Deletion on Chromosome 1 Causes Malignant Peripheral Nerve Sheath Tumors and Carcinomas in Zebrafish
Source: PLoS One. 2015 Dec 22;10(12):e0145178. doi: 10.1371/journal.pone.0145178 (PMC4687860; doi:10.1371/journal.pone.0145178)

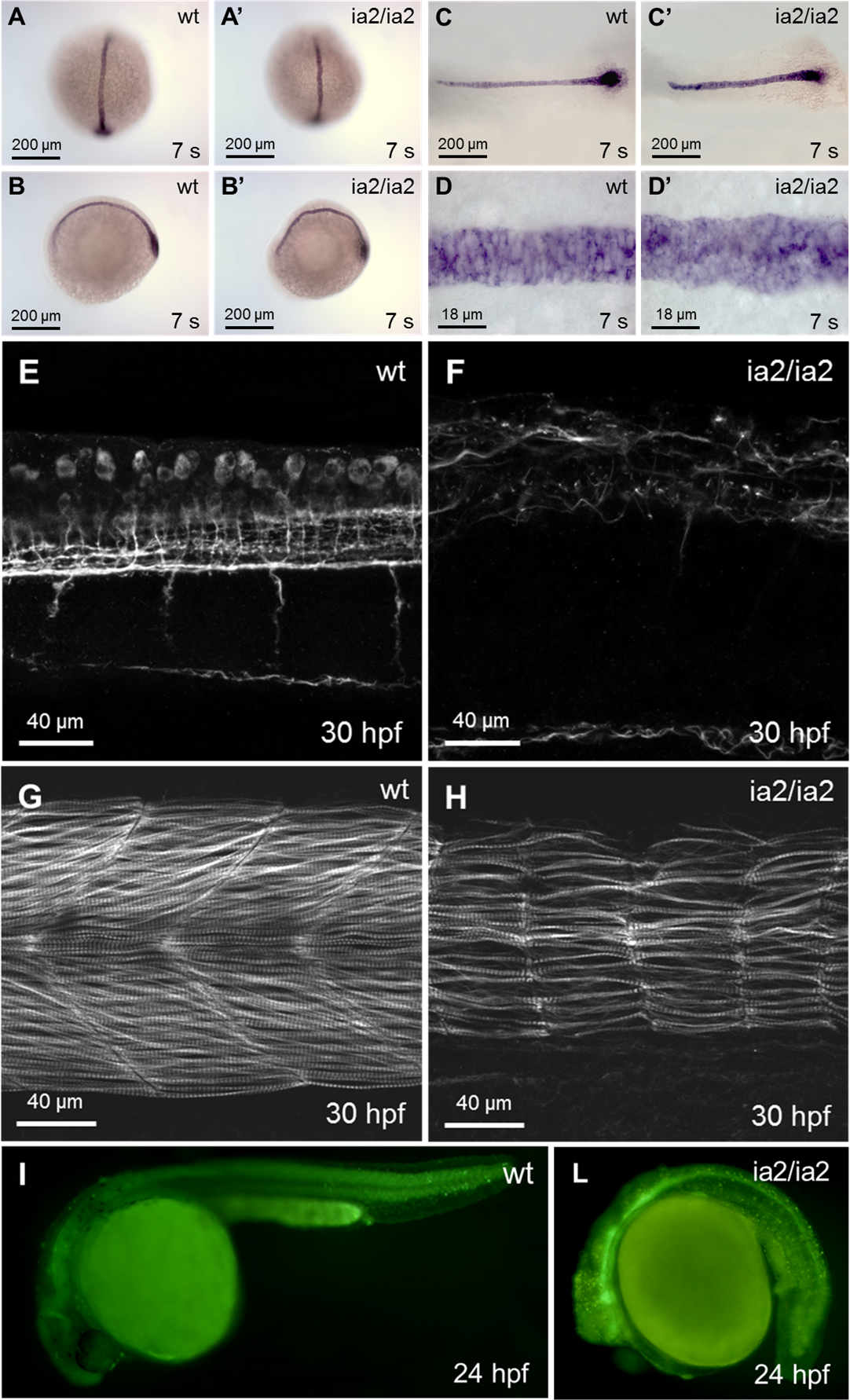

Supplement: S1 Fig — (A-D) Notochord alterations are evidenced by in situ hybridization for notail already at 7 somites stage. Mutant notochord is shorter and wider with respect to the wild-type (C, C’). Notochord bending (A-B’) and the morphological abnormalities of notochord structure and vacuolated cells (C-D’) are also apparent. (E, F) Axonal microtubules staining with anti-acetylated tubulin antibody. Confocal Z-stack projection; trunk side view. The mutant shows a highly disorganized axonal network. (G, H) Rhodamine-phalloidin staining. Confocal Z-stack projection; trunk side view. In the mutant the muscle fibers are strongly reduced in number, with an overall dramatic decline in muscle fibers mass. (I, L) Acridine orange staining. Massive cell death is evident in ia2/ia2 mutant, particularly in the regions of the head and the tail corresponding to the morphologically most altered districts. The intense GFP fluorescence of the neural tube visible in the mutant is that characteristic of the Tg(-8.5nkx2.2a:GFP) ia2 transgenic line. (TIF) [file pone.0145178.s001.tif]
