# Supplementary material for: A GFP-Tagged Gross Deletion on Chromosome 1 Causes Malignant Peripheral Nerve Sheath Tumors and Carcinomas in Zebrafish
Source: PLoS One. 2015 Dec 22;10(12):e0145178. doi: 10.1371/journal.pone.0145178 (PMC4687860; doi:10.1371/journal.pone.0145178)
Supplement: S2 Table — (PDF) [file pone.0145178.s003.pdf]

| | GENE | PRODUCT | Mb |
|---|---|---|---|
| 1 | timm10 | mitochondrial import inner membrane translocase subunit Tim10 | 43,69 |
| 2 | unc93b1 | protein unc-93 homolog B1 | 43,69 |
| 3 | cryba1l2 | crystallin beta A1 like 2 | 43,72 |
| 4 | crybb1 l2 | crystallin beta B1 like 2 | 43,74 |
| 5 | rtn4rl2a | reticulon 4 receptor-like 2 a precursor | 43,79 |
| 6 | zdhhc5a | zinc finger DHHC-type containing 5a | 43,84 |
| 7 | tmx2b | thioredoxin-related transmembrane protein 2-B precursor | 43,87 |
| 8 | med19b | mediator of RNA polymerase II transcription subunit 19-B | 43,88 |
| 9 | slc43a1b | large neutral amino acids transporter small subunit 3 | 43,89 |
| 10 | si:dkey-28b4.7 | DNA polymerase subunit delta-4 | 44,01 |
| 11 | p2rx3b | P2X purinoceptor 3 | 44,04 |
| 12 | si:dkey-9i23.4 | uncharacterized protein LOC571855 precursor | 44,07 |
| 13 | si:dkey-9i23.5 | uncharacterized protein LOC571862 precursor | 44,08 |
| 14 | ndufs8a | NADH dehydrogenase [ubiquinone] iron-sulfur protein 8 mitochondrial | 44,11 |
| 15 | zgc:165520 | uncharacterized protein LOC571872 | 44,13 |
| 16 | kdm2aa | lysine (K)-specific demethylase 2B | 44,14 |
| 17 | si:dkey-9i23.15 | uncharacterized protein LOC555790 precursor | 44,22 |
| 18 | tmem176 | transmembrane protein 176 | 44,23 |
| 19 | si:dkey-9i23.16 | uncharacterized protein LOC571915 | 44,24 |
| 20 | vps37c | vacuolar protein sorting-associated protein 37C | 44,25 |
| 21 | dnaja11 | dnaJ homolog subfamily A member 1 | 44,30 |
| 22 | rps6 | 40S ribosomal protein S6 | 44,34 |
| 23 | zgc:56382 | uncharacterized protein LOC406670 precursor | 44,47 |
| 24 | zgc:111983 | uncharacterized protein LOC550501 precursor | 44,47 |
| 25 | ddx39aa | DEAD (Asp-Glu-Ala-Asp) box polypeptide 39Aa | 44,50 |
| 26 | emp1 | epithelial membrane protein 1 | 44,63 |
| 27 | zgc:101679 | factor VIII intron 22 protein | 44,64 |
| 28 | si:ch211-243a20.3 | uncharacterized protein LOC100151507 precursor | 44,66 |
| 29 | grin2bb | glutamate [NMDA] receptor subunit epsilon-2 | 44,68 |
| 30 | atf7ip | activating transcription factor 7-interacting protein 1 | 44,88 |
| 31 | sept3 | neuronal-specific septin-3 | 44,93 |
| 32 | serhl | serine hydrolase-like protein | 44,96 |
| 33 | trappc5 | trafficking protein particle complex subunit 5 | 44,97 |
| 34 | mcoln1 | mucolipin 1 | 44,98 |
| 35 | si:ch211-214c7.5 | UPF0575 protein C19orf67 homolog | 45,05 |
| 36 | map2k7 | dual specificity mitogen-activated protein kinase kinase 7 | 45,15 |
| 37 | lipt1 | lipoyltransferase 1 mitochondrial | 45,23 |
| 38 | arhgef7b | Rho guanine nucleotide exchange factor (GEF) 7b | 45,27 |
| 39 | sox1b | transcription factor Sox-1b | 45,50 |
| 40 | tubgcp | gamma-tubulin complex component 3 | 45,71 |
| 41 | si:busm1-105l16.2 | uncharacterized protein LOC368709 isoform 2 | 45,81 |
| 42 | zgc:174719 | uncharacterized protein LOC100137112 | 45,81 |
| 43 | cab39l | calcium-binding protein 39-like | 45,91 |
| 44 | cdadc1 | cytidine and dCMP deaminase domain-containing protein 1 | 45,93 |
| 45 | rcbtb1 | RCC1 and BTB domain-containing protein 1 | 45,94 |
| 46 | arl11 | ADP-ribosylation factor-like protein 11 | 45,96 |
| 47 | ebpl | emopamil-binding protein-like | 45,97 |
| 48 | kpna3 | importin subunit alpha-3 | 45,98 |
| 49 | spryd7a | chromosome 13 open reading frame 1 | 46,02 |
| 50 | mir16b | microRNA | 46,03 |
| 51 | mir15a-1 | microRNA | 46,03 |
| 52 | si:ch73-160h15.3 | leukemia-associated protein 7 | 46,14 |
| 53 | agpat3 | 1-acyl-sn-glycerol-3-phosphate acyltransferase gamma | 46,15 |
| 54 | pdxkb | pyridoxal kinase | 46,20 |
| 55 | itsn1 | intersectin-1 | 46,31 |
| 56 | cryzl1 | quinone oxidoreductase-like protein 1 | 46,40 |
| 57 | setd4 | SET domain-containing protein 4 | 46,42 |
| 58 | zgc:162351 | uncharacterized protein LOC100038770 precursor | 46,43 |
| 59 | si:ch211-287j19.6 | uncharacterized protein LOC565708 precursor | 46,43 |
| 60 | mhc1zda | major histocompatibility complex class I ZDA precursor | 46,43 |
| 61 | zgc:136614 | uncharacterized protein LOC664757 precursor | 46,45 |

| | | | |
|---|---|---|---|
| 62 | zgc:111893 | uncharacterized protein LOC556341 precursor | 46,45 |
| 63 | mhc1zba | major histocompatibility complex class I ZE like precursor | 46,46 |
| 64 | mhc1zha | major histocompatibility complex class I ZHA precursor | 46,47 |
| 65 | cbr1 | carbonyl reductase [NADPH] 1 | 46,47 |
| 66 | morc3b | MORC family CW-type zinc finger 3b | 46,49 |
| 67 | bcl9 | B-cell CLL/lymphoma 9 protein | 46,70 |
| 68 | cx41.8 | connexin 41.8 | 46,72 |
| 69 | cx44.1 | gap junction alpha-8 protein | 46,75 |
| 70 | fstl1a | follistatin-like 1a precursor | 46,77 |
| 71 | lrrc58a | leucine rich repeat containing 58 | 46,79 |
| 72 | obfc1 | CST complex subunit STN1 | 46,81 |
| 73 | sh3pxd2aa | SH3 and PX domain-containing protein 2A | 46,89 |
| 74 | neurl1aa | E3 ubiquitin-protein ligase NEURL1 | 47,01 |
| 75 | cfap58 | coiled-coil domain-containing protein 147 | 47,14 |
| 76 | cxcl18b | chemokine (C-X-C motif) ligand 18b precursor | 48,58 |
| 77 | msxb | homeobox protein MSH-B | 48,71 |
| 78 | si:dkeyp-80c12.7 | uncharacterized protein LOC557882 | 48,73 |
| 79 | taf5 | transcription initiation factor TFIID subunit 5 | 48,77 |
| 80 | si:dkeyp-80c12.10 | up-regulated during skeletal muscle growth protein 5 | 48,79 |
| 81 | pdcd11 | protein RRP5 homolog | 48,79 |
| 82 | zgc:175214 | ring finger protein 122-like | 48,84 |
| 83 | si:ch211-281g13.5 | probable E3 ubiquitin-protein ligase HERC4-like | 48,88 |
| 84 | zgc:163136 | uncharacterized protein LOC100073325 | 48,89 |
| 85 | si:ch211-281g13.4 | si:ch211-281g13.4 | 48,90 |
| 86 | col17a1a | collagen type XVII alpha 1a | 48,92 |
| 87 | slkb | non-coding RNA | 48,96 |
| 88 | tsga10 | testis-specific gene 10 protein | 49,01 |
| 89 | lef1 | lymphoid enhancer-binding factor 1 | 49,17 |
| 90 | hadh | hydroxyacyl-coenzyme A dehydrogenase mitochondrial | 49,27 |
| 91 | cyp2u1 | cytochrome P450 2U1 | 49,28 |
| 92 | sgms2 | phosphatidylcholine:ceramide cholinephosphotransferase 2 | 49,29 |
| 93 | gstcd | glutathione S-transferase C-terminal domain-containing protein | 49,31 |
| 94 | npnt | nephronectin precursor | 49,44 |
| 95 | tbck | TBC domain-containing protein kinase-like protein | 49,55 |
| 96 | aimp1 | aminoacyl tRNA synthase complex-interacting multifunctional protein 1 | 49,65 |
| 97 | dkk2 | dickkopf-related protein 2 precursor | 49,75 |
| 98 | papss1 | bifunctional 3'-phosphoadenosine 5'-phosphosulfate synthase 1 | 49,84 |
| 99 | pkd2 | polycystin-2 | 49,90 |
| 100 | abcg2d | ATP-binding cassette sub-family G member 2 | 49,92 |
| 101 | ppm1k | protein phosphatase 1K (PP2C domain containing) | 49,95 |
| 102 | herc3 | probable E3 ubiquitin-protein ligase HERC3 | 50,00 |
| 103 | zgc:154142 | uncharacterized protein LOC555481 | 50,15 |
| 104 | otx1a | homeobox protein OTX1 A | 50,28 |
| 105 | mdh1aa | malate dehydrogenase 1Aa NAD (soluble) | 50,33 |
| 106 | mdh1aa | malate dehydrogenase 1Aa NAD (soluble) | 50,33 |
| 107 | mdh1aa | malate dehydrogenase 1Aa NAD (soluble) | 50,33 |
| 108 | ugp2a | UDP-glucose pyrophosphorylase 2a | 50,35 |
| 109 | spast | spastin | 50,38 |
| 110 | dpy30 | non-coding RNA | 50,40 |
| 111 | dpy30 | protein dpy-30 homolog | 50,40 |
| 112 | psen2 | presenilin-2 | 50,41 |
| 113 | srd5a2a | steroid-5-alpha-reductase alpha polypeptide 2a | 50,42 |
| 114 | jag1a | protein jagged-1a precursor | 50,46 |
| 115 | btbd3a | BTB (POZ) domain containing 3 | 50,55 |
| 116 | esf1 | ESF1 homolog | 50,56 |
| 117 | ndufaf5 | NADH dehydrogenase [ubiquinone] 1 alpha subcomplex assembly factor 5 precursor | 50,58 |
| 118 | actr2a | actin-related protein 2-A | 50,76 |
| 119 | meis4.1a | myeloid ecotropic viral integration site 4.1a | 50,84 |
| 120 | etaa1 | Ewing tumor-associated antigen 1 | 50,89 |
| 121 | ppp3r1b | protein phosphatase 3 (formerly 2B) regulatory s1ubunit B alpha isoform b | 50,91 |
| 122 | cnrip1a | cannabinoid receptor interacting protein 1a | 50,95 |
| 123 | zgc:165656 | uncharacterized protein LOC100126009 | 50,98 |
| 124 | prokr1a | prokineticin receptor 2 | 51,04 |
| 125 | snrpb2 | U2 small nuclear ribonucleoprotein B'' | 51,07 |

| | | | |
|---|---|---|---|
| 126 | rnaseh2a | ribonuclease H2 subunit A | 51,08 |
| 127 | prdx2 | peroxiredoxin-2 | 51,09 |
| 128 | junba | transcription factor jun-B | 51,10 |
| 129 | dand5 | DAN domain family member 5 precursor | 51,19 |
| 130 | rad23aa | UV excision repair protein RAD23 homolog A | 51,48 |
| 131 | rnf11a | ring finger protein 11a | 51,50 |
| 132 | asna1 | ATPase asna1 | 51,50 |
| 133 | rab3da | RAB3D member RAS oncogene family a | 51,54 |
| 134 | pld6 | mitochondrial cardiolipin hydrolase | 51,57 |
| 135 | cnn1a | uncharacterized protein LOC567396 | 51,58 |
| 136 | zgc:194101 | uncharacterized protein LOC798684 | 51,80 |
| 137 | si:ch211-217k17.10 | si:ch211-217k17.10 | 51,81 |
| 138 | si:ch211-217k17.7 | uncharacterized protein LOC559409 | 51,82 |
| 139 | glb1 | beta-galactosidase precursor | 51,83 |
| 140 | cldnd1b | claudin domain containing 1b | 51,85 |
| 141 | LOC554386 | uncharacterized protein LOC554386 | 51,85 |
| 142 | acy3.2 | aspartoacylase-2B | 51,86 |
| 143 | acy3.1 | aspartoacylase-2A | 51,86 |
| 144 | tacr3l | tachykinin receptor 3-like | 51,88 |
| 145 | abca1a | ATP-binding cassette sub-family A member 1 | 51,93 |
| 146 | aptx | aprataxin | 52,02 |
| 147 | osbp | oxysterol-binding protein 1 | 52,03 |
| 148 | patl1 | protein PAT1 homolog 1 | 52,13 |
| 149 | smarca5 | SWI/SNF-related matrix-associated actin-dependent regulator of chromatin subfamily A m | 52,16 |
| 150 | il15 | interleukin 15 | 52,60 |
| 151 | znf330 | zinc finger protein 330 | 52,63 |
| 152 | rnf150 | RING finger protein 150 precursor | 52,65 |
| 153 | ucp1 | mitochondrial uncoupling protein 3 | 52,74 |
| 154 | elmod2 | ELMO/CED-12 domain containing 2 precursor | 52,76 |
| 155 | zgc:66455 | uncharacterized protein LOC393502 precursor | 52,82 |
| 156 | b3gnt2a | UDP-GlcNAc:betaGal beta-1 3-N-acetylglucosaminyltransferase 2a | 52,84 |
| 157 | commd1 | COMM domain-containing protein 1 | 52,86 |
| 158 | cct4 | T-complex protein 1 subunit delta | 53,07 |
| 159 | pus10 | pseudouridylate synthase 10 | 53,08 |
| 160 | crfb17 | interferon gamma receptor 1 precursor | 53,18 |
| 161 | ltv1 | protein LTV1 homolog | 53,21 |
| 162 | capn9 | calpain-9 | 53,22 |
| 163 | taf5l | TAF5-like RNA pol II p300/CBP-associated factor-associated factor 65 kDa subunit | 53,25 |
| 164 | nup133 | nuclear pore complex protein Nup133 | 53,26 |
| 165 | acta1a | actin gamma-enteric smooth muscle | 53,29 |
| 166 | dla | delta-like protein A | 53,35 |
| 167 | tekt4 | tektin-4 | 53,94 |
| 168 | sh:ch211-202h22.9 | si:ch211-202h22.9 | 53,95 |
| 169 | si:ch211-202h22.8 | lipopolysaccharide-induced tumor necrosis factor-alpha factor-like | 53,96 |
| 170 | gprc5bb | G-protein coupled receptor family C group 5 member B-like precursor | 54,00 |
| 171 | exosc1 | 3'-5' exoribonuclease CSL4 homolog | 54,02 |
| 172 | pgam1b | phosphoglycerate mutase 1b | 54,03 |
| 173 | cdc25d | cell division cycle 25 homolog d | 54,04 |
| 174 | pi4k2a | phosphatidylinositol 4-kinase type 2-alpha | 54,06 |
| 175 | zfyve27 | zinc finger FYVE domain containing 27 | 54,21 |
| 176 | golga 7ba | golgin A7 family member Ba | 54,23 |
| 177 | crtac1a | cartilage acidic protein 1a precursor | 54,26 |
| 178 | khsrp | far upstream element-binding protein 2 | 54,28 |
| 179 | zgc:136864 | UPF0390 protein zgc136864 | 54,33 |
| 180 | vps54 | vacuolar protein sorting-associated protein 54 | 54,33 |
| 181 | lgalsla | galectin-related protein | 54,40 |
| 182 | aftpha | aftiphilin | 54,42 |
| 183 | mb | myoglobin | 54,46 |
| 184 | pane1 | centromere protein M | 54,48 |
| 185 | zgc:158803 | uncharacterized protein LOC100038767 | 54,49 |
| 186 | mri1 | methylthioribose-1-phosphate isomerase | 54,51 |
| 187 | nanos3 | nanos homolog 3 | 54,57 |
| 188 | si:ch211-286b5.5 | guanine nucleotide-binding protein GI/GS/GO gamma-5 subunit | 54,59 |
| 189 | zgc:172106 | uncharacterized protein LOC100136844 precursor | 54,62 |

| | | | |
|---|---|---|---|
| 190 | dnajb1b | DnaJ (Hsp40) homolog subfamily B member 1 | 55,07 |
| 191 | tecrb | trans-2 3-enoyl-CoA reductase | 55,07 |
| 192 | ndufb7 | NADH dehydrogenase [ubiquinone] 1 beta subcomplex subunit 7 | 55,10 |
| 193 | zgc:136908 | transitional endoplasmic reticulum ATPase-like | 55,11 |
| 194 | asf1bb | histone chaperone asf1b-B | 55,17 |
| 195 | prkacab | cAMP-dependent protein kinase catalytic subunit gamma | 55,20 |
| 196 | c3a.6 | complement C3-H1-like precursor | 55,36 |
| 197 | c3a.1 | complement component c3a precursor | 55,46 |
| 198 | crb3b | crumbs homolog 3b | 55,51 |
| 199 | zgc:65894 | uncharacterized protein LOC335798 | 55,55 |
| 200 | c3a.2 | complement component c3a duplicate 2 precursor | 55,56 |
| 201 | c3a.3 | complement component c3a duplicate 3 precursor | 55,61 |
| 202 | mir722 | microRNA | 55,69 |
| 203 | slc25a10 | mitochondrial dicarboxylate carrier | 55,75 |
| 204 | zgc:171452 | uncharacterized protein LOC100126110 | 55,79 |
| 205 | sc:d0284 | uncharacterized protein LOC100135433 precursor | 56,28 |
| 206 | b3gntl1 | UDP-GlcNAc:betaGal beta-1 3-N-acetylglucosaminyltransferase-like protein 1 | 56,63 |
| 207 | zgc:171470 | uncharacterized protein LOC565848 precursor | 56,78 |
| 208 | tspan34 | uncharacterized protein LOC751731 | 56,81 |
| 209 | si:ch211-114l13.9 | uncharacterized protein LOC796649 | 57,26 |
| 210 | nxnl1 | nucleoredoxin-like protein 1 | 57,27 |
| 211 | si:ch211-114l13.7 | procollagen galactosyltransferase 1 precursor | 57,28 |
| 212 | trim35-1 | uncharacterized protein LOC553197 | 57,30 |
| 213 | caspb | caspase b | 57,32 |
| 214 | caspbl | uncharacterized protein LOC566185 | 57,37 |
| 215 | zgc:194906 | uncharacterized protein LOC100170839 | 57,71 |
| 216 | zgc:174890 | uncharacterized protein LOC792547 | 57,80 |
| 217 | zgc:174928 | uncharacterized protein LOC792701 | 57,81 |
| 218 | slc27a1b | solute carrier family 27 (fatty acid transporter) member 1 | 57,87 |
| 219 | zgc:172091 | uncharacterized protein LOC100136869 | 57,89 |
| 220 | sid4 | secreted immunoglobulin domain 4 precursor | 57,97 |
| 221 | tmed1b | interleukin 1 receptor-like 1 ligand precursor | 58,13 |
| 222 | dnm2l | dynamin-2 | 58,16 |
| 223 | zgc:171687 | uncharacterized protein LOC100126131 precursor | 58,22 |
| 224 | trip10b | thyroid hormone receptor interactor 10b | 58,22 |
| 225 | pin1 | peptidyl-prolyl cis-trans isomerase NIMA-interacting 1 | 58,29 |
| 226 | zgc:173915 | uncharacterized protein LOC100126122 precursor | 58,31 |
| 227 | mfap4 | microfibrillar-associated protein 4 precursor | 58,31 |
| 228 | lpar2b | lysophosphatidic acid receptor 2b | 58,42 |
| 229 | akap8l | A-kinase anchor protein 8-like | 58,44 |
| 230 | zgc:153247 | probable glutamate--tRNA ligase mitochondrial precursor | 58,45 |
| 231 | eri2 | ERI1 exoribonuclease 2 | 58,48 |
| 232 | thumpd1 | THUMP domain-containing protein 1 | 58,48 |
| 233 | zgc:76872 | uncharacterized protein LOC403007 | 58,51 |
| 234 | mvb12a | multivesicular body subunit 12A | 58,53 |
| 235 | olfm2b | olfactomedin 2 like | 58,54 |
| 236 | col5a3b | collagen type V alpha 3b precursor | 58,55 |
| 237 | rdh8b | retinol dehydrogenase 8b | 58,59 |
| 238 | parn | poly(A)-specific ribonuclease PARN | 58,61 |
| 239 | cyp3a65 | cytochrome P450 family 3 subfamily A polypeptide 65 | 58,63 |
| 240 | zgc:77358 | transcription factor Sp6 | 58,83 |
| 241 | wfikkn1 | WAP kazal immunoglobulin kunitz and NTR domain-containing protein precursor | 58,86 |
